# Novel Catalytically-Inactive PII Metalloproteinases from a Viperid Snake Venom with Substitutions in the Canonical Zinc-Binding Motif

**DOI:** 10.3390/toxins8100292

**Published:** 2016-10-12

**Authors:** Erika Camacho, Libia Sanz, Teresa Escalante, Alicia Pérez, Fabián Villalta, Bruno Lomonte, Ana Gisele C. Neves-Ferreira, Andrés Feoli, Juan J. Calvete, José María Gutiérrez, Alexandra Rucavado

**Affiliations:** 1 Instituto Clodomiro Picado, Facultad de Microbiología, Universidad de Costa Rica, San José 11501, Costa Rica; ecamachoum@gmail.com (E.C.); teresa.escalante@ucr.ac.cr (T.E.); fvillalta85@gmail.com (F.V.); bruno.lomonte@ucr.ac.cr (B.L.); boch31@gmail.com (A.F.); jose.gutierrez@ucr.ac.cr (J.M.G.); 2 Instituto de Biomedicina de Valencia, Consejo Superior de Investigaciones Científicas, Valencia 46010, Spain; libia.sanz@ibv.csic.es (L.S.); aperez@ibv.csic.es (A.P.); jcalvete@ibv.csic.es (J.J.C.); 3 Laboratório de Toxinologia, Instituto Oswaldo Cruz, Fiocruz, Rio de Janeiro 21040-900, Brazil; anagextra@gmail.com; 4 Departamento de Biotecnología, Universidad Politécnica de Valencia, Valencia 46022, Spain

**Keywords:** snake venom metalloproteinases, PII SVMP homologues, disintegrin domain, zinc-binding motif, hemorrhagic activity, platelet aggregation, proteinase activity

## Abstract

Snake venom metalloproteinases (SVMPs) play key biological roles in prey immobilization and digestion. The majority of these activities depend on the hydrolysis of relevant protein substrates in the tissues. Hereby, we describe several isoforms and a cDNA clone sequence, corresponding to PII SVMP homologues from the venom of the Central American pit viper *Bothriechis lateralis*, which have modifications in the residues of the canonical sequence of the zinc-binding motif HEXXHXXGXXH. As a consequence, the proteolytic activity of the isolated proteins was undetectable when tested on azocasein and gelatin. These PII isoforms comprise metalloproteinase and disintegrin domains in the mature protein, thus belonging to the subclass PIIb of SVMPs. PII SVMP homologues were devoid of hemorrhagic and in vitro coagulant activities, effects attributed to the enzymatic activity of SVMPs, but induced a mild edema. One of the isoforms presents the characteristic RGD sequence in the disintegrin domain and inhibits ADP- and collagen-induced platelet aggregation. Catalytically-inactive SVMP homologues may have been hitherto missed in the characterization of snake venoms. The presence of such enzymatically-inactive homologues in snake venoms and their possible toxic and adaptive roles deserve further investigation.

## 1. Introduction

Snake venom metalloproteinases (SVMPs) are abundant components in the venoms of advanced snakes of the superfamily Colubroidea and play key roles in venom toxic and digestive actions [1,2,3]. Together with the ADAMs and ADAMTs, SVMPs are classified within the M12 family of metalloproteinases (subfamily M12B or reprolysin) [1], which, in turn, belong to the metzincin superfamily of these proteinases. This superfamily is characterized by a canonical zinc-binding sequence (HEXXHXXGXXH) followed by a Met-turn [4].

SVMPs have been classified into three classes on the basis of the domain composition (PI, PII and PIII). All contain a signal sequence, a prodomain and a metalloproteinase domain. In the case of the PI class, the mature protein has only the metalloproteinase domain, while the PII class has, in addition to the catalytic domain, a disintegrin (Dis) domain, which may be proteolytically processed, giving rise to a free disintegrin, that in most cases hosts the canonical RGD motif [5]. In turn, PIII class SVMPs have disintegrin-like (Dis-like) and cysteine-rich (Cys-rich) domains following the metalloproteinase domain. In some PIII SVMPs, two additional C-type lectin-like subunits are covalently linked to the cysteine-rich domain. Within each one of these SVMP classes, there are several subclasses that vary in their post-translational processing and quaternary structure [1,6]. SVMPs play key biological roles for snakes, both as digestive enzymes and as toxicity factors, since they are responsible for local and systemic hemorrhage, blistering and dermonecrosis, myonecrosis and coagulopathies, in addition to exerting a pro-inflammatory role [2]. Most of the biological effects of SVMPs depend on their catalytic activity, particularly on their capacity to hydrolyze extracellular matrix components and coagulation factors. However, other functions are associated with the non-metalloproteinase domains, such as the action of disintegrins and disintegrin-like sequences on platelet aggregation [1,2,7,8].

SVMPs constitute a model of evolution and neofunctionalization of multigene families [9]. The evolutionary history of these toxins, which has been characterized by domain loss, gene duplication, positive selection and neofunctionalization, started with the recruitment of an ADAM (ADAM 7, ADAM 28 and/or ADAM decysin 1), followed by the loss of C-terminal domains characteristic of ADAMs, resulting in the formation of P-III SVMPs [10,11]. After that, a duplicated SVMP PIII gene evolved by positive selection into a PII SVMP via loss of the Cys-rich domain. Then, PI evolved by disintegrin domain loss, with further neofunctionalization of the metalloproteinase domain to play diverse biological roles [6,10,11]. PIII SVMPs are present in all advanced snake families, whereas PI and PII SVMPs occur only in the family Viperidae [2,6]. This accelerated and complex evolutionary process has generated a great functional diversity in this group of enzymes and the expression of multiple, structurally-distinct SVMP isoforms in snake venoms [6,12,13].

Many representatives of PII SVMPs undergo a post-translational modification by which the disintegrin domain is released from the precursor protein [1,6]. However, in two subclasses of PII SVMPs, i.e., PIIb and PIIc, this proteolytic cleavage does not occur, and the mature proteins are comprised by the metalloproteinase and the Dis domain held together [6]. In addition, PIIc SVMPs occur as dimers, whereas PIIb are monomers. Despite the great diversification of SVMPs, to the best of our knowledge, there are no reports on enzymes with mutations in the canonical zinc-binding motif of the active site, and all SVMPs described to date are catalytically active.

In the course of the characterization of SVMPs from the venom of the arboreal pit viper *Bothriechis lateralis*, a species distributed in Central America [14], a fraction containing internal sequences characteristic of SVMPs, but being devoid of proteinase and hemorrhagic activities, was isolated. The characterization of this fraction and the cDNA cloning of a related isoform showed the presence of modifications in the residues constituting the zinc-binding motif characteristic of the reprolysin group of metalloproteinases. This study describes the isolation and characterization of these SVMP variants and discusses some biological implications of their presence in snake venoms.

## 2. Results

### 2.1. Purification of SVMP BlatPII

**Figure 1:**
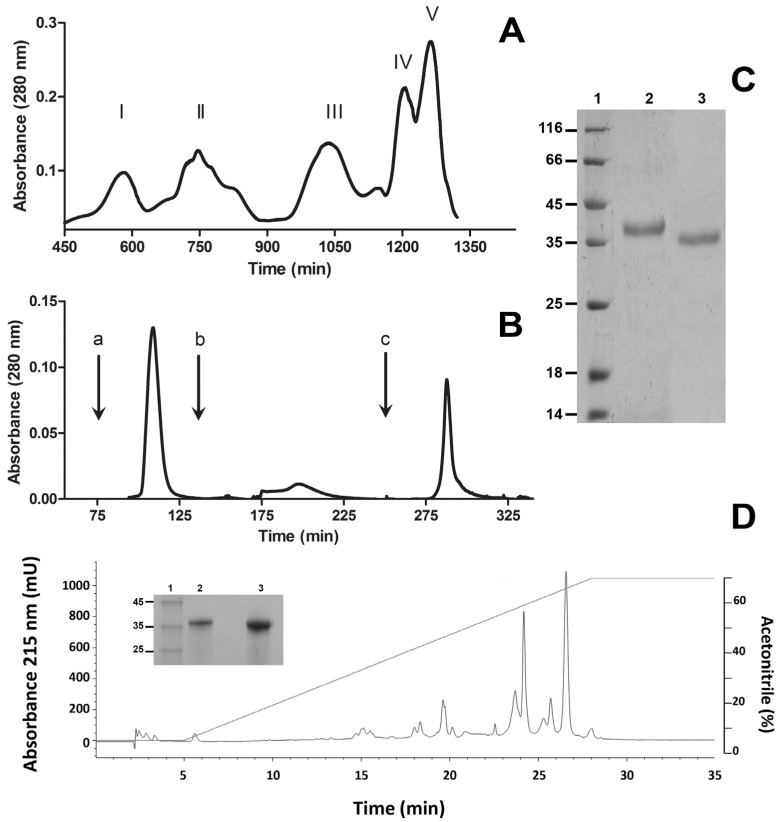
Isolation of BlatPII from *B. lateralis* venom. Venom was fractionated by ion-exchange chromatography on diethylaminoethyl (DEAE)-Sepharose (**A**); and hydrophobic interaction chromatography on phenyl sepharose (**B**); as described in the Materials and Methods. Arrows inserted in (**B**) correspond to the addition of 100 mL of 0.01 M phosphate buffer, pH 7.8, containing 1 M NaCl (a); 100 mL of 0.01 M phosphate buffer, pH 7.8 (b); and 100 mL of deionized water (c). Fraction II from the hydrophobic interaction chromatography is a SVMP devoid of hemorrhagic activity and was named BlatPII. (**C**) SDS-PAGE of the purified protein under reducing (Lane 2) and non-reducing (Lane 3) conditions. (**D**) BlatPII was separated into two main peaks by RP-HPLC. Red line corresponds to acetonitrile gradient (see the text for details). SDS-PAGE under reducing conditions of Peak 1 (Lane 2) and Peak 2 (Lane 3) is shown in the insert of (**D**). Lane 1 in gels of (**C**) and the insert of (**D**) corresponds to molecular mass standards (kDa).

A PII SVMP, hereby named BlatPII, was purified from the venom of *B. lateralis* by two chromatographic steps. First, ion-exchange chromatography on diethylaminoethyl (DEAE)-Sepharose yielded five protein fractions (Figure 1A). Then, Peak III, which showed hemorrhagic activity, was fractionated by phenyl sepharose chromatography (Figure 1B). Fraction II of the latter chromatography was devoid of hemorrhagic activity, but had sequences characteristic of SVMPs by mass spectrometric analysis. When run on SDS-PAGE, it showed bands of 36 kDa and 39 kDa, under non-reducing and reducing conditions, respectively (Figure 1C), indicating that it is a monomeric protein containing intramolecular disulfide bond(s). It stained positive in the carbohydrate detection test, i.e., it is a glycoprotein (not shown). Reversed-phase HPLC analysis demonstrated that the protein, apparently homogeneous on SDS-PAGE, can be separated into two peaks (Figure 1D) with molecular masses of 35,409 ± 248 Da and 36,715 ± 249 Da, estimated by MALDI-TOF mass spectrometry.

### 2.2. Determination of Internal Peptide Sequences

Mass spectrometry analysis and Edman *N*-terminal degradation of peptides obtained by digestion of RP-HPLC Peaks 1 and 2 with different proteinases identified several sequences characteristic of the metalloproteinase and disintegrin domains of SVMPs (Table 1). Sequences having two different patterns of substitutions in the canonical sequence of the zinc-binding motif of SVMPs (HEXXHXXGXXH) were found. One of them (HELGHNLGIHQ) has a Gln instead of a characteristic His, whereas the other (HDLGHNLCIDH) has two substitutions, i.e., an Asp instead of Glu and a Cys replacing a Gly. These sequences were found in peptides having variations in other internal sequences, hence evidencing the presence of several isoforms within these two basic patterns of substitutions in the zinc-binding motif (Table 1). Representative high-resolution MS/MS spectra of the above referred peptides are shown in the Supplementary Material (Figures S1–S5). All fragmentation patterns were automatically interpreted by PEAKS De Novo software (version 8, Bioinformatics Solutions Inc., Waterloo, Canada, 2016). Only sequence results with very high confidence (average local confidence (ALC) ≥99% and local confidence score for each amino acid ≥98%) were considered. The sequence RGD, characteristic of many disintegrins, was observed in a few peptides derived from proteins eluted in the HPLC Peak 2 (Table 1). The high-resolution MS/MS spectrum of one of these peptides is shown in the Supplementary Material (Figure S6). For this specific peptide, the de novo ALC was 91%, and the local confidence score for each amino acid ranged from 59% to 99%. All peptides derived from proteins from HPLC Peaks 1 and 2 identified by automatic de novo sequencing and showing ALC ≥ 99% were aligned against the BlatPII-c sequence using PepExporer, a similarity-driven tool (Figures S7 and S8). Considering a minimum identity of 75%, the sequence coverage obtained for BlatPII-c was 22.1% and 49.1%, respectively, for peptides from HPLC Peak 1 and Peak 2.

### 2.3. Cloning of a P-II SVMP

**Figure 2:**
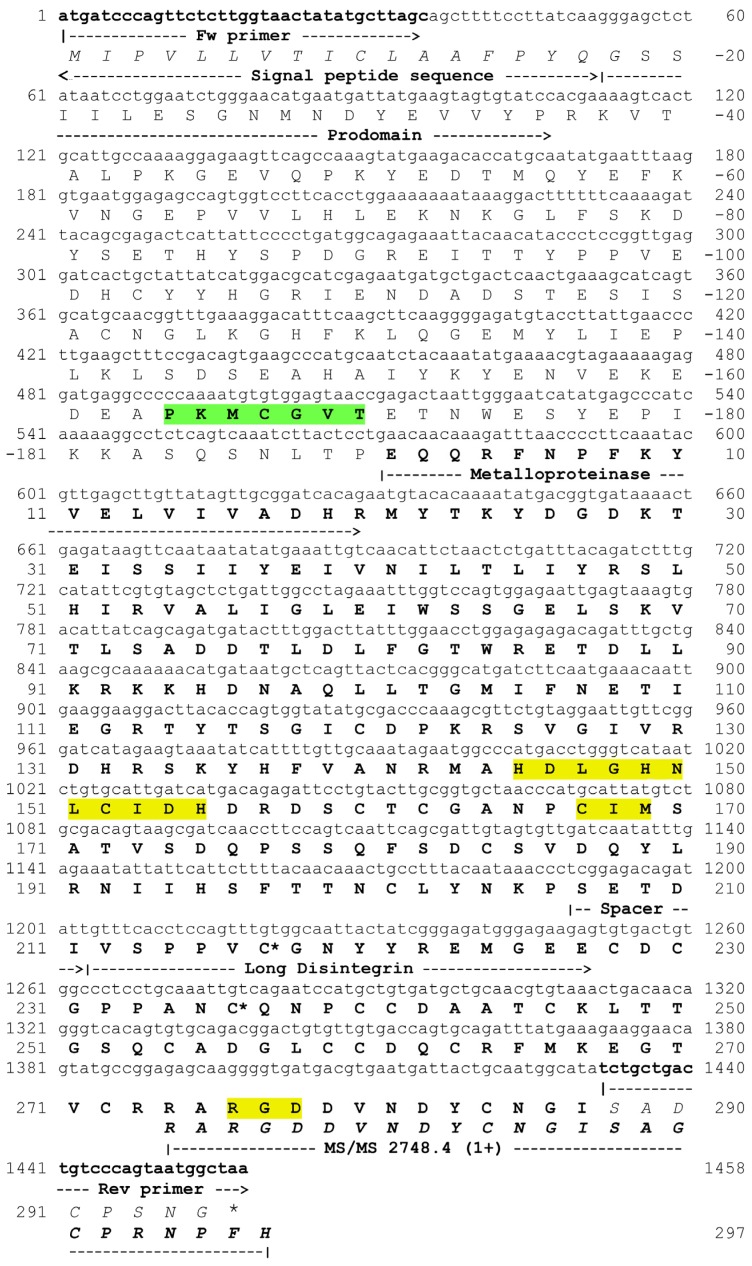
cDNA sequence and deduced amino acid sequence of BlatPII-c. The deduced amino acid sequence of the mature protein is depicted in bold letters and is preceded by the pro-domain, which contains the sequence PKMCGVT characteristic of the Cys switch (highlighted in green). Sequences corresponding to signal peptide, pro-domain, metalloproteinase domain, spacer region and the disintegrin domain are indicated. The sequences corresponding to the mutated zinc-binding motif and the sequence CIM (Cys-Ile-Met) of the Met-turn are highlighted in yellow, as well as the sequence RGD in the disintegrin domain. Cysteine residues proposed to be related to the lack of proteolytic processing of the disintegrin from the metalloproteinase domain are labeled with asterisks. Primers used to PCR-amplify the cDNA clone and the C-terminal amino acid sequence gathered by MS/MS analysis of a tryptic peptide are shown.

The PCR amplified a 1458-bp cDNA sequence (Figure 2), with a precursor protein sequence including a 17-residue *N*-terminal signal peptide, a pre-pro-domain (residues −18–−190, including the canonical cysteine switch motif (PKMCGVT)), followed by a mature protein comprising a 206-amino acid metalloproteinase domain, a short spacer sequence (residues 207–211) and a long PII disintegrin domain (212–297) containing the characteristic RGD motif. Noteworthy, the metalloproteinase domain contains the CIM sequence characteristic of the Met-turn of metzincins [4] and a mutated zinc-binding motif with the sequence HDLGHNLCIDH, instead of the canonical HEXXHXXGXXH. This sequence is identical to the mutated catalytic site of some of the internal sequences determined in proteins eluted in Peak 2 of the RP-HPLC separation (Table 1). When comparing the complete sequence of the clone with the peptide sequences identified in RP-HPLC peaks (Table 1), there were differences in some residues. Thus, this cDNA-deduced amino acid sequence is likely to correspond to another isoform having the same substitutions in the canonical catalytic site found in some of the isolated protein isoforms. The protein coded by the cDNA clone is hereby named BlatPII-c. The BlatPII-c clone presents two Cys residues, corresponding in the mature protein to residues at positions 217 and 236. The presence of these Cys residues identifies this domain as a member of the long-chain disintegrin subfamily [13,15] and has been suggested to underlay the lack of proteolytic processing of the disintegrin domain in some PII SVMPs. Therefore, the data suggest that BlatPII-c belongs to the subclass PIIb of SVMPs, i.e., monomeric PII containing metalloproteinase and disintegrin domains in the mature protein [16] (Figure 2). The sequence of this clone was deposited in GenBank under Accession Number KU885992.

### 2.4. Proteolytic Activity on Azocasein and Gelatin

**Figure 3:**
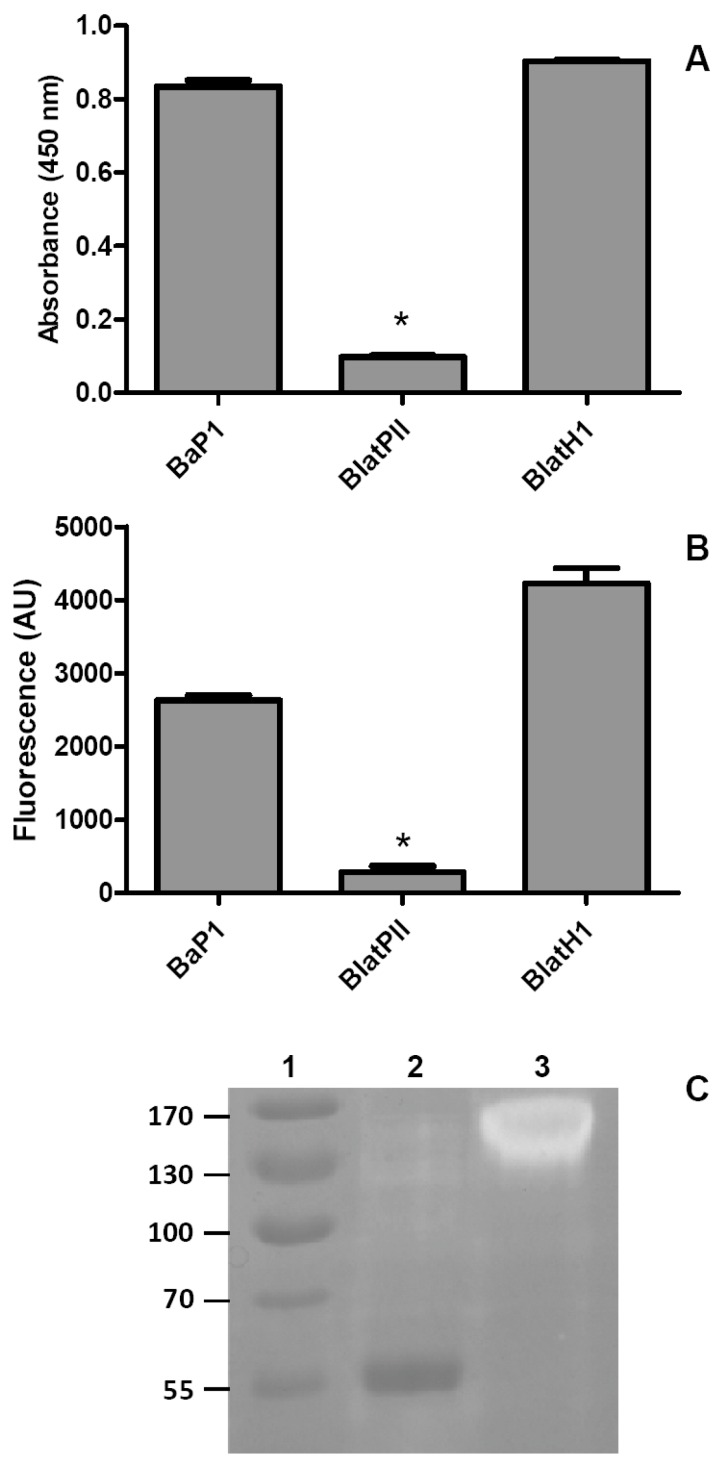
Evaluation of the proteolytic activity of BlatPII and other SVMPs on azocasein and gelatin. (**A**) BlatPII (5.5 µM) was incubated with azocasein (see the Materials and Methods for details). For comparison, an equimolar concentration of PI SVMP BaP1, from the venom of *Bothrops asper*, and of the hemorrhagic PII SVMP BlatH1, from the venom of *Bothriechis lateralis*, were also tested. PBS was used as a negative control; (**B**) Quantification of the gelatinolytic activity of the SVMP by a fluorescent commercial kit (EnzCheck^®^ protocol Gelatinase/Collagenase Assay Kit, Molecular Probes, Life Technologies, Eugene, OR, USA). One-point-six micrograms of BlatPII were incubated with 20 µg of the fluorescent gelatin; equimolar quantities of BaP1 and BlatH1 were used as positive controls; samples were incubated at room temperature for 6 h. Fluorescence intensity was measured in the BioTek Synergy HT microplate reader using the absorption filter at 495 nm and the emission filter at 515 nm. * *p* < 0.05 when compared with BaP1 and BlatH1. In (**A**,**B**), the signal of BlatPII was not significantly different when compared to the controls without enzyme (*p* > 0.05); (**C**) Gelatin zymography: 2.5 µg of BlatPII (Lane 2) and 2.5 µg of BlatH1 (Lane 3) were separated on SDS-PAGE under non-reducing conditions. Lane 1 corresponds to molecular mass standards. The dark band observed in Lane 2 corresponds to BlatPII, whose migration in SDS-PAGE-gelatin gels is delayed as compared to SDS-PAGE gels without gelatin.

Proteolytic activity on these two substrates was tested with the fraction BlatPII, which contains isoforms that can be separated only by RP-HPLC. Since the solvents used for this separation denature the enzymes, it was not possible to assess the proteolytic activity of the RP-HPLC peaks separately. Under the experimental conditions used, BlatPII did not show proteolytic activity on the two substrates tested. In contrast, both BaP1 and BlatH1 SVMPs, which are active proteinases, hydrolyzed azocasein and gelatin (Figure 3).

### 2.5. Hemorrhagic, Edema-Forming, Coagulant and Platelet Aggregation Inhibitory Activities

**Figure 4:**
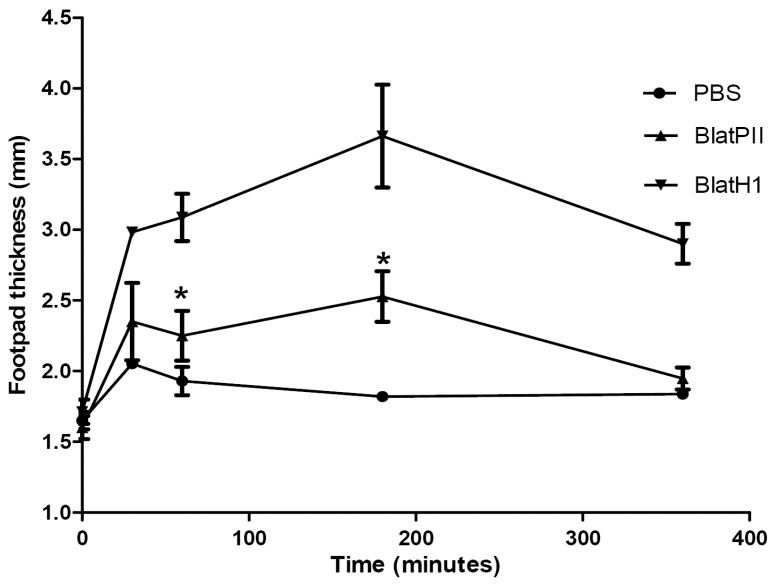
Edema-forming activity of BlatPII. Groups of four CD-1 mice were injected subcutaneously in the right footpad with 5 µg of BlatPII, in a volume of 50 µL PBS. Controls were injected with 50 µL PBS only. The paw thickness was measured using a low pressure spring caliper, as a quantitative index of edema, before injection and at 30, 60, 180 and 360 min after injection. Edema was calculated as the percentage of increase in the paw thickness of the right foot injected with SVMP as compared to the left foot; in parallel, 5 µg of BlatH1 were used as a positive control. BlatH1 induced significantly higher (*p* < 0.05) edema than BlatPII at all time intervals, whereas BlatPII induced a significant edema, as compared to the control, only at 60 and 180 min (* *p* < 0.05).

**Figure 5:**
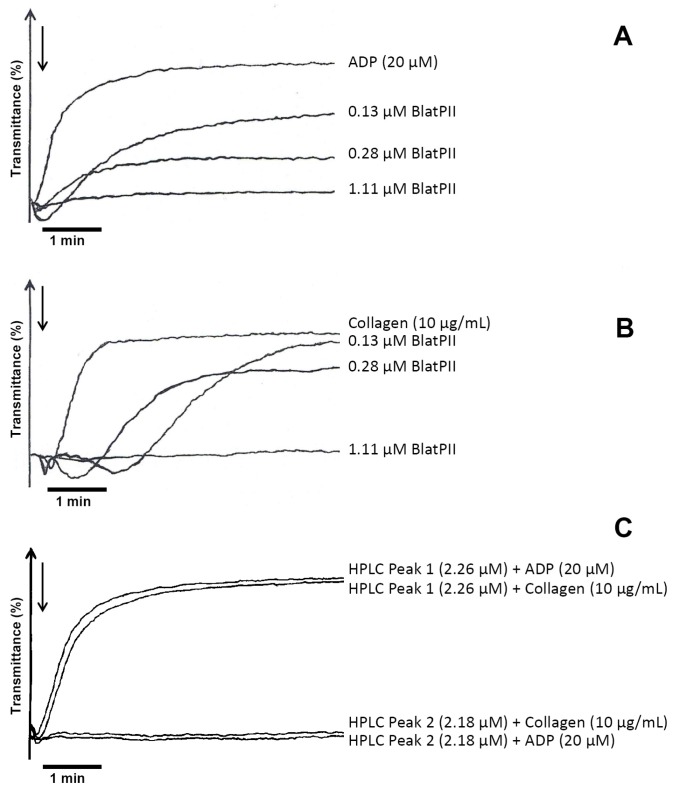
Inhibition of platelet aggregation by BlatPII and RP-HPLC Peaks 1 and 2. Various concentrations of BlatPII were tested using ADP (**A**) or collagen (**B**) as agonists. (**C**) Inhibition of platelet aggregation by HPLC Peak 1 and Peak 2. Results presented correspond to one experiment representative of three different independent experiments.

BlatPII did not exert local hemorrhagic activity when injected in the skin of mice at a dose of 100 µg, nor did it show coagulant activity on human plasma. Likewise, the protein did not induce pulmonary hemorrhage after i.v. injection of 100 µg in mice. On the other hand, BlatPII demonstrated a low edema-forming activity (Figure 4), and inhibited ADP- and collagen-mediated aggregation of human platelets in PRP, with estimated IC_50_s of 0.25 and 0.33 µM, respectively (Figure 5A,B). In the case of the peaks obtained by RP-HPLC separation, Peak 1 did not induce the inhibition of platelet aggregation, while Peak 2 inhibited platelet aggregation using ADP and collagen as the agonists (Figure 5C).

### 2.6. Three-Dimensional Model of the Active Site of BlatPII-c

**Figure 6:**
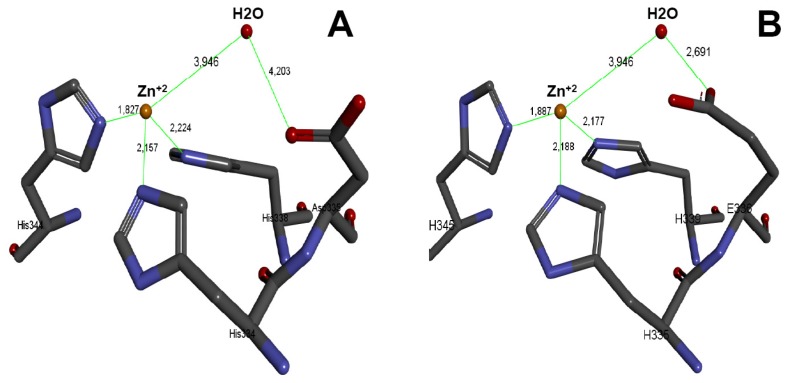
3D model of the active site HDLGHNLCIDH. Using the Discovery Studio Program Version 3.5.0.12158 (Accelrys Software Inc, San Diego, CA, USA), a 3D model of the active site of BlatPII was generated (**A**). Determination of distances in the predicted model was made using the coordinates of the zinc atom and the water molecule from the crystallographic structure of VAP-1 from venom of *Crotalus atrox* (**B**).

As described above, the clone codes for the sequence HDLGHNLCIDH instead of the characteristic canonical site HEXXHXXGXXH at the enzyme active site. Three-dimensional structures, based on homology models, were generated using the Modeller method available in the Accelrys Discovery Studio 3.5 software [17]. The vascular apoptosis-inducing protein 1 (VAP-1) (PDB Code 2ERQ) was used as a template. After aligning the generated model of the BlatPII-c with the VAP-1 crystal structure, it was shown that the substitution of Glu for Asp causes an increase in the distance between residue Asp335 and the water molecule (4.203 Å), both of which play a key role in the hydrolysis of the peptide bond. In the case of VAP-1, a PIII SVMP from the venom of *Crotalus atrox*, which has the characteristic active site with Glu, the distance between the water molecule and Glu is 2.691 Å (Figure 6). A PIII SVMP (VAP-1) was used as a model, because no PII SVMP has been crystallized, and the sequence identity percentage between these toxins is acceptable (55%) for the modeling program.

## 3. Discussion

In contrast to the SVMPs described so far, the PII SVMP homologues described in the present study have the peculiarity of being devoid of proteinase activity. To the best of our knowledge, this constitutes the first case of a snake venom protein having a SVMP structural scaffold, but being devoid of proteolytic activity. Mass spectrometric and molecular analyses of the isoforms of BlatPII underscore the presence of mutations in the canonical sequence of the zinc-binding motif characteristic of the active site of SVMPs. In the canonical sequence (HEXXHXXGXXH), the catalytic zinc is coordinated by the three His residues, and a water molecule plays the role of a fourth ligand and is clamped between the catalytic Glu of the motif and the metal, forming a trigonal pyramidal coordination sphere around the zinc [4]. In the catalytic process, the water molecule is added to the peptidic bond by a nucleophilic attack, in which the carbonyl group of the scissile peptide bond is polarized by the active site zinc. In such a mechanism, the zinc-bound water molecule mediates between this carbonyl group and the catalytic Glu residue, giving rise to the acquisition of nucleophilicity by the water molecule, necessary for attacking the carbonyl carbon of the scissile peptide bond, resulting in a tetra-coordinate transition state. The catalytic Glu then presumably serves as a proton shuttle to the cleavage products [4,18,19]. Thus, it is likely that the substitutions of these highly-conserved catalytic residues occurring in the isoforms of BlatPII drastically affect one or more steps in this catalytic process. The presence of catalytically-inactive SVMP homologues in snake venoms needs to be further investigated since the isolation of SVMPs is usually followed by testing proteinase or toxic activities of the chromatographic fractions; this approach does not detect catalytically-inactive variants. It is therefore recommended that the search for this type of inactive SVMP homologues is based on either immunological detection of components cross-reacting with SVMPs or on the proteomic identification of these variants, as performed in our study.

In contrast to SVMPs, where no examples of enzymatically-inactive variants have been described before, mutations in the catalytic site have been reported in the case of the other branch of the reprolysin group of metalloproteinases, i.e., the ADAMs [1,20,21]. Some ADAMs have completely lost the sequence of the zinc-binding motif [1,21,22,23,24]. In other cases, such as ADAMDEC-1 [20] and ADAM-7 [21,25], substitutions in this canonical motif have been described, with variable impact on the proteolytic activity of these proteins. On the other hand, the detection of several mutated isoforms of BlatPII and the presence of variations in the internal sequences determined highlight the existence of several isoforms in this venom within the two basic mutated patterns of the zinc-binding motif.

Two basic patterns of substitutions in the canonical sequence of the zinc-binding motif were found in our study. In one case, an Asp substitutes the Glu and a Cys substitutes a Gly, whereas in the other, a Gln substitutes the third His in the canonical sequence. It is therefore proposed that these substitutions drastically affect the catalytic machinery in these PII SVMP homologues. A 3D modeling of the active site, using the sequence of BlatPII-c, showed that the substitution of Glu for Asp causes an increase of the distance between the Asp335 residue and the water molecule, as compared to the distance between Glu335 and water in a catalytically-active variant. The increment of this distance may affect the activation of the water molecule, thus precluding the nucleophilic attack of the peptide bond. On the other hand, the substitution of a Gly by Cys might also bear structural consequences that affect the catalytic machinery. The elucidation of the 3D structure of BlatPII-c will certainly provide an explanation for the described effect in proteolytic activity.

The lack of hemorrhagic and coagulant activities in BlatPII is probably related to the loss of enzymatic activity, since these effects have been associated with proteolysis in catalytically-active SVMPs. The observation that BlatPII induces mild edema is compatible with a study that described edema-forming activity of a fragment constituted by the Dis-like and Cys-rich domains [26]. HPLC peak 2 inhibits platelet aggregation induced by ADP and collagen, and as expected, sequences found in HPLC Peak 2 present the characteristic RGD motif in the disintegrin domain. Since this sequence is associated with the ability of many disintegrins to bind α_IIB_β_3_ integrin in the platelet membrane, causing inhibition of aggregation [27,28,29], it is suggested that it is responsible for the effect observed in platelets.

The existence of catalytically-inactive PII SVMP homologues in the venom of *B. lateralis* is intriguing from the adaptive standpoint, since most of the described biological roles of SVMPs, associated with both prey immobilization and digestion, are dependent on the proteolytic degradation of diverse substrates, usually extracellular matrix components or plasma proteins [2]. BlatPII isoforms are devoid of hemorrhagic and coagulant effects, in agreement with their lack of proteinase activity. The only biological effects shown by BlatPII were a mild edema-forming effect and inhibition of platelet aggregation, which may be due to the presence of RGD in the disintegrin domain and may play a role in the overall toxicity of the venom by affecting this aspect of hemostasis, although this requires experimental demonstration. Alternatively, the presence of BlatPII in the venom may reveal a neutral evolutionary process by which these catalytically-inactive homologues are expressed in the venom gland without playing a significant trophic role. Many components have been described in snake venoms that have not been shown to play a biological role. Nevertheless, it would be relevant to explore the possible toxicity of these isoforms by assessing a wider set of effects and also by studying their toxicity in prey other than mice, since adult *B. lateralis* also feeds on birds and bats in addition to rodents [14].

In the wider context of hydrolytic enzymes, examples of catalytically-inactive homologues of phospholipases A_2_ (PLA_2_) and of serine proteinases have been described in snake venoms. In the case of PLA_2_ homologues, substitutions of key residues in the catalytic machinery have resulted in the loss of enzymatic activity. However, many catalytically-inactive PLA_2_ homologues exert toxic effects, such as myotoxicity and pro-inflammatory activities, owing to the appearance of ‘toxic sites’ in some regions of the surface of these molecules [30]. Likewise, serine proteinase homologues have been reported, which are characterized by substitutions in the catalytic triad His, Asp, Ser [31,32]. In a serine proteinase isolated from the venom of *Trimeresurus jerdonii*, the replacement of His-43 by Arg-43 at the catalytic triad has been associated with the loss of proteolytic activity [32].

In conclusion, cDNA encoding for a PII SVMP and several isoforms of PII SVMP homologues with a mutated catalytic site occur in the venom of the arboreal viperid snake *B. lateralis*, which contains a high percentage of SVMPs (55.1%) [33]. Such mutations in the canonical sequence of the zinc-binding motif of these enzymes are likely to be responsible for the lack of enzymatic activity observed. To the best of our knowledge, this is the first case of catalytically-inactive SVMP homologues described in snake venoms. The possible biological role, if any, of these SVMP variants remains unknown, although one of them is able to inhibit ADP- and collagen-induced platelet aggregation in vitro. These components expand the wide set of SVMP variants in viperid snake venoms. The search for other enzymatically-inactive SVMP homologues in venoms is a relevant task in order to ascertain how frequent they are and to discover new possible biological roles for this group of venom components.

## 4. Materials and Methods

### 4.1. Venom and Toxins

A pool of venom obtained from at least 20 adult specimens of *B. lateralis* collected in various locations of Costa Rica, and maintained at the Serpentarium of Instituto Clodomiro Picado, was used. Venom was lyophilized and stored at −20 °C. Venom solutions were prepared immediately before each experiment. In some experiments, BlatH1, a PII SVMP isolated from the venom of *B. lateralis* [34], and BaP1, a PI SVMP from the venom of *Bothrops asper* [35], were used for comparative purposes.

### 4.2. Purification of SVMP

One hundred milligrams of *B. lateralis* venom were dissolved in 5 mL of 0.01 M phosphate buffer, pH 7.8, and centrifuged at 500× *g* for 10 min. The supernatant was loaded onto a DEAE sepharose column (column 10 cm in length × 2 cm internal diameter) previously equilibrated with the same buffer. After eluting the unbound material, a linear NaCl gradient (0–0.4 M) was applied, and fractions were collected at a flow rate of 0.3 mL/min. The fraction containing SVMPs, identified on the basis of hemorrhagic activity in mice, was then adjusted to 1 M NaCl and applied to a phenyl sepharose hydrophobic interaction column (6 cm × 1.5 cm i.d.). Unbound proteins were eluted with 100 mL of 0.01 M phosphate buffer, pH 7.8, containing 1 M NaCl. Then, 100 mL of 0.01 M phosphate buffer, pH 7.8, were applied, and finally, the chromatographic separation was finished by the addition of 100 mL of deionized water. The eluted fractions were analyzed by SDS-PAGE, hemorrhagic activity and mass spectrometry analysis of tryptic peptides. A peak of 39 kDa devoid of hemorrhagic activity, but showing amino acid sequences corresponding to SVMPs, was isolated after the application of 100 mL of 0.01 M phosphate buffer, pH 7.8, and was further characterized in this study; it was named BlatPII. The peak obtained after the application of water has been previously characterized as a dimeric PII hemorrhagic SVMP, known as BlatH1 [34]. Homogeneity and molecular mass were determined by SDS-polyacrylamide gel electrophoresis (SDS-PAGE) under reducing and non-reducing conditions, using 12% polyacrylamide gels [36].

### 4.3. HPLC Separation of Isoforms

One hundred micrograms of BlatPII were loaded on a Thermo AQUASIL C4 column (150 mm × 4.6 mm i.d.), and the reversed-phase HPLC fractionation was made on an Agilent 1200 instrument (Agilent Technologies, Santa Clara, CA, USA) at a flow of 0.5 mL/min, with detection set at 215 nm. The solvent system was 0.1% trifluoroacetic acid in H_2_O (Solvent A) and 0.1% trifluoroacetic acid in acetonitrile (Solvent B). The gradient program began with 0% Solvent B for 5 min and was then ramped to 70% Solvent B at 300 min, followed by 70% Solvent B for 5 min.

### 4.4. Mass Spectrometry Analyses

#### 4.4.1. MALDI-TOF/TOF

Mass determination of the whole protein was performed by mixing 0.5 μL of saturated sinapinic acid (in 50% acetonitrile, 0.1% trifluoroacetic acid) and 0.5 μL of sample, which were spotted onto an OptiTOF-384 plate, dried and analyzed in positive linear mode on an Applied Biosystems 4800-Plus MALDI-TOF/TOF instrument (Applied Biosystems, Framingham, MA, USA). Mass spectra were acquired with delayed extraction in the *m*/*z* range of 20,000–130,000, at a laser intensity of 4200 and 500 shots per spectrum. Protein identification and amino acid sequencing of peptides obtained by digestion with trypsin, chymotrypsin or endoproteinase Glu-C were carried out by CID tandem mass spectrometry. Protein bands were excised from Coomassie Blue R-250-stained gels (12% polyacrylamide) following SDS-PAGE run under either reducing or non-reducing conditions and were reduced with dithiothreitol, alkylated with iodoacetamide and digested with sequencing-grade bovine trypsin (Sigma-Aldrich Co, St. Louis MO, USA), chymotrypsin (GBiosciences, St. Louis, MO, USA) or endoproteinase Glu-C (V8 protease) (GBiosciences, St. Louis, MO, USA), on an automated processor (ProGest, Digilab, Marlborough, MA, USA) overnight at 37 °C, according to the instructions of the manufacturer. The peptide mixtures were then analyzed by MALDI-TOF-TOF. Mixtures of 0.5 μL of sample and 0.5 μL of saturated α-cyano-4-hydroxycinnamic acid (in 50% acetonitrile, 0.1% trifluoroacetic acid) were spotted, dried and analyzed in positive reflector mode. Spectra were acquired using a laser intensity of 3000 and 1625 shots per spectrum, after external MS and MS/MS calibration with CalMix-5 standards (ABSciex, Framingham, MA, USA) spotted on the same plate. Up to 10 precursor peaks were selected from each MS spectrum for automated collision-induced dissociation MS/MS spectra acquisition at 2 kV (500 shots/spectrum, laser intensity of 3000). The spectra were analyzed using ProteinPilot v.4.0.8 and the Paragon^®^ algorithm (ABSciex) against the UniProt/SwissProt database (Serpentes; downloaded 12 January 2016) for protein identification at a confidence level of 99%. Subsequently, sequences were manually inspected to confirm amino acid sequences de novo.

#### 4.4.2. nLC-MS/MS

RP-HPLC Peaks 1 and 2 were dissolved in 0.4 M ammonium bicarbonate/8 M urea, reduced in 10 mM dithiothreitol at 37°C for 3 h, alkylated in 25 mM of iodoacetamide, followed by final quenching step in 4.5 mM DTT. The last two steps were performed at room temperature, in the dark, for 15 min each. After diluting the urea to 1 M, trypsin (Promega, Madison, WI, USA) was added (enzyme to substrate ratio 1:50 *w*/*w*) and the hydrolysis proceeded for 18 h at 37°C. Trifluoroacetic acid was then added to a final concentration of 1% (*v*/*v*), followed by sample desalting on Poros R2 microcolumns. Peptides were completely dried in a vacuum centrifuge, followed by re-suspension with 1% formic acid in water. Peptide quantitation (2 μL aliquots) was based on the UV absorbance at 280 nm on a NanoDrop 2000 spectrophotometer (Thermo Scientific, Waltham, MA, USA) (1 Absorbance unit = 1 mg/mL, considering 1 cm path length). Peptides (~1 μg) were submitted to a reversed phase nanochromatography (Ultimate 3000 system, Thermo Scientific Dionex, Sunnyvale, CA, USA) hyphenated to a quadrupole Orbitrap mass spectrometer (Q Exactive Plus, Thermo Scientific, Waltham, MA, USA). For desalting and concentration, samples were first loaded at 2 μL/min onto a home-made capillary guard column (2 cm × 100 μm i.d.) packed with 5 μm, 200 Å Magic C18 AQ matrix (Michrom Bioresources, Auburn, CA, USA). Peptide fractionation was performed at 200 nL/min on an analytical column (30 cm × 75 μm i.d.) with a laser pulled tip (~5 μm), packed with 1.9 μm ReproSil-Pur 120 C18-AQ (Dr. Maisch). The following mobile phases were used: (A) 0.1% formic acid in water; (B) 0.1% formic acid in acetonitrile. Peptides were eluted with a gradient of 2%–40% B over 162 min, which was increased to 80% in 4 min, followed by a washing step at this concentration for 2 min before column re-equilibration.

Using data-dependent acquisition, up to 12 most intense precursor ions in each survey scan (excluding singly-charged ions and ions with unassigned charge states) were selected for higher energy collisional dissociation (HCD) fragmentation with 30% normalized collision energy. The following settings were used: (a) full MS (profile mode): 70,000 resolution (FWHM at *m*/*z* 200), Automatic Gain Control (AUC) AGC target 1 × 10^6^, maximum injection time 100 ms, scan range 300–1500 *m*/*z*; (b) dd-MS/MS (centroid mode): 17,500 resolution, AGC target 5 × 10^4^, maximum injection time 50 ms, isolation window 2.0 *m*/*z* (with 0.5 *m*/*z* offset), dynamic exclusion 60 s. The spray voltage was set to 1.9 kV with no sheath or auxiliary gas flow and with a capillary temperature of 250 °C. Data were acquired in technical triplicates using the Xcalibur software (Version 3.0.63, Thermo Fisher Scientific, Waltham, MA, USA). To avoid cross-contamination, two blank injections were run before the analysis of each biological replicate. The mass spectrometer was externally calibrated using a calibration mixture that was composed of caffeine, peptide MRFA and Ultramark 1621, as recommended by the instrument manufacturer.

PEAKS (version 8, Bioinformatics Solutions Inc., Waterloo, KW, Canada, 2016) was used for de novo sequencing analysis, using the following parameters: fixed cysteine modification (carbamidomethylation); 10 ppm peptide mass error tolerance; 0.02 Da fragment mass tolerance. Only de novo sequences showing ≥99% ALC (average of local confidence) were exported and submitted to sequence alignment against the mature BlatPII-c clone sequence using the algorithm PepExplorer [37]. The following parameters were used in the similarity-driven analysis: 75% minimum identity, minimum of 6 residues per peptide, substitution matrix PAM30MS.

### 4.5. Determination of Internal Peptide Sequences by Edman Degradation

The fractions obtained by reversed-phase HPLC were dried and reconstituted in 20 µL of 0.4 M NH_4_HCO_3_, 8 M urea. Then, 5 µL of 100 mM dithiothreitol were added, and the solution was incubated for 3 h at 37 °C. After that, 400 mM of iodoacetamide were added and incubated at room temperature for 15 min, protected from light. Then, 130 µL of deionized water were added, and digestion was performed with 2 µg trypsin (Sigma-Aldrich Co, St. Louis, MO, USA), overnight at 37 °C. The reaction was stopped with 20 µL of 1% (*v*/*v*) trifluoroacetic acid. Peptides were separated by reversed-phase HPLC on an Agilent 1200 instrument (Agilent Technologies, Santa Clara, CA, USA) in a Thermo AQUASIL C18 column (4.6 × 150 mm) at a flow of 0.5 mL/min, with detection at 215 nm. The solvent system was 0.1% trifluoroacetic acid in H_2_O (Solvent A) and 0.1% trifluoroacetic acid in acetonitrile (Solvent B). The gradient program started with 5% Solvent B for 5 min and was then raised to 75% Solvent B at 50 min, and 75% Solvent B was maintained for 5 min. *N*-terminal sequences of isolated peptides were determined on a Shimadzu Biotech PPSQ 33A instrument according to the manufacturer´s instruction. These tryptic peptides were also used for MALDI-TOF/TOF MS analysis.

### 4.6. Detection of Carbohydrates

Two-point-five micrograms of purified SVMP were separated by 12% SDS-PAGE under reducing and non-reducing conditions. The gel was stained using a Pro-Q Emerald 300 Glycoprotein Kit (Molecular Probes, Eugene, OR, USA), as described by the manufacturer.

### 4.7. PCR Amplification of SVMPs cDNA

An adult specimen of *B. lateralis*, kept at the Serpentarium of Instituto Clodomiro Picado, was sacrificed 3 days after venom extraction, and its venom glands were dissected out for total RNA isolation. After homogenization of the venom glands, the total RNA was extracted using the RNeasy Mini Kit (Qiagen, Hilden, Germany), following the manufacturer´s instructions. The first strand of cDNA was reverse-transcribed using the RevertAid H Minus First Strand cDNA Synthesis kit, according to the manufacturer's (ThermoScientific, Waltham, MA, USA) protocol, using the Qt-primer 5′-CCAGTGAGCAGAGTGACGAGGACTCGAGCTCAAGC(T)17-3′. cDNAs coding for PII SVMP sequences were amplified by PCR using the following primers: Forward (Fw) primer 5′-ATGATCCCAGTTCTCTTGGTAACTATATGCTTAGC-3′ (corresponding to a conserved sequence in the pro-peptide of a metalloproteinase precursor; Figure 2), and reverse (Rev) primer, 5′-AGCCATTACTGGGACAGTCAGCAG-3′, coding for the C-terminal amino acid sequence of BlatPII, obtained by mass spectrometry analysis (Figure 2). The PCR program was as follows: initial denaturation step (94 °C for 2 min), 35 cycles of denaturation (94 °C for 45 s), annealing (55 °C for 45 s), extension (72 °C for 120 s) and final extension (72 °C for 10 min). The PCR products were separated by 1% agarose electrophoresis, and the fragments with the expected molecular mass were purified using Illustra GFXTM PCR DNA and Gel Band Purification Kit (GE Healthcare, Life Sciences, Uppsala, Sweden). The purified sequences were cloned in a pGEM-T vector (Promega, Madison, WI, USA), which were used to transform *Escherichia coli* DH5α cells (Novagen, Darmstadt, Germany) by using an Eppendorf 2510 electroporator following the manufacturer's instructions. Positive clones, selected by growing the transformed cells in LB (Luria-Bertani medium) (Thermo Fisher Scientific, Waltham, MA, USA) broth containing 10 μg/mL of ampicillin, were confirmed by PCR-amplification using M13 primers of the pGEM-T vector. The inserts of positive clones were isolated using kit Wizard (Promega) and sequenced on an Applied Biosystems Model 377 DNA sequencer.

### 4.8. Proteolytic Activity on Azocasein

The proteinase activity of the SVMP on azocasein was assessed according to [38]. For comparative purposes, the PI SVMP BaP1 and the dimeric PII SVMP BlatH1 were used.

### 4.9. Proteolytic Activity on Gelatin

Proteolytic activity on gelatin was assessed by two methods: gelatin zymography and using a fluorescent commercial kit (EnzCheck^®^ protocol Gelatinase/Collagenase Assay Kit, Molecular Probes, Life Technologies, Eugene, OR, USA). For the zymography, the method described by Herron et al. [39], as modified by Rucavado et al. [40], was used. Briefly, 2.5 µg of the SVMP were separated on a 10% SDS-PAGE containing 0.5 mg/mL of Type A gelatin (Sigma Chemical Co., St Louis, MO, USA). The same amount of BlatH1 was included as a positive control. After electrophoresis, gels were incubated with zymography substrate buffer overnight at 37 °C. Then, the gel was stained with 0.5% Coomassie Blue R-250 in acetic acid:isopropyl alcohol:water (1:3:6) and destained with distilled water to visualize gelatinolytic bands. In order to quantify the gelatinolytic activity of the SVMP, the commercial kit EnzCheck^®^ was used following the manufacturer’s instructions. Various concentrations of the SVMP, dissolved in 100 µL of reaction buffer, were incubated with 20 µg of the fluorescent gelatin substrate in a 96-well microplate. Samples were incubated at room temperature for 6 h and protected from light. Samples were analyzed in triplicate; a reagent blank was included. BlatH1 and BaP1 were included as positive controls. Fluorescence intensity was measured in the BioTek Synergy HT microplate reader using the absorption filter at 495 nm and the emission filter at 515 nm.

### 4.10. Local and Systemic Hemorrhagic Activities

Local hemorrhagic activity was determined by the mouse skin test [41]. Groups of four CD-1 mice (18–20 g) were intradermally (i.d.) injected in the ventral abdominal region with different doses of *B. lateralis* SVMP, dissolved in 100 µL of PBS. After 2 h, animals were sacrificed by CO_2_ inhalation. The presence of hemorrhagic areas in the inner side of the skin was determined. The experimental protocols involving the use of animals in this study were approved by the Institutional Committee for the Care and Use of Laboratory Animals (CICUA) of the University of Costa Rica. Systemic hemorrhagic activity was tested as described by Escalante et al. [42]. Groups of four CD-1 mice were intravenously (i.v.) injected, in the tail vein, with various doses of the SVMP, dissolved in 100 µL PBS. After one hour, mice were sacrificed by an overdose of a ketamine/xylazine mixture. The thoracic cavity was opened, and the presence of hemorrhagic spots was assessed by macroscopic observation of the lungs. Lungs of injected animals were dissected out and fixed in formalin solution for processing and embedding in paraffin. Then, sections were stained with hematoxylin-eosin for microscopic observation.

### 4.11. Edema-Forming Activity

The edema-forming activity was assessed according to the method of Lomonte et al. [43]. Groups of four CD-1 mice were injected subcutaneously (s.c.) in the right footpad with 5 µg of SVMP, dissolved in 50 µL PBS. In other mice, the SVMP BlatH1 was used as a positive control. Control mice were injected with PBS under otherwise identical conditions. The paw thickness was measured using a low pressure spring caliper before injection and at 0, 30, 60, 180 and 360 min after injection. Edema was calculated as the percentage of increase in the paw thickness of the right foot injected with SVMP as compared to the thickness before injection.

### 4.12. Coagulant Activity

Coagulant activity was determined as described by [44], with the following modifications: various doses of SVMP, dissolved in 25 µL PBS, were added to 0.25 mL of citrated human plasma, previously incubated for 5 min at 37 °C. Coagulant activity was monitored for 15 min at 37 °C. PBS was used as a negative control.

### 4.13. Platelet Aggregation Inhibitory Activity

Platelet-rich plasma (PRP) was obtained from adult healthy human volunteers, as described [45]. Two hundred twenty five microliters of PRP were pre-warmed at 37 °C for 5 min. The SVMP, dissolved in 25 µL of sterile saline solution, was added to PRP, and the mixture incubated for 5 min. After that, either ADP (20 µM final concentration) or collagen (10 µg/mL final concentration) (Helena Laboratories, Beaumont, TX, USA) was added to the PRP-SVMP mixture, and platelet aggregation was recorded for 5 min using an AggRAM analyzer (Helena Laboratories, Beaumont, Texas, TX, USA) at a constant spin rate of 600 rpm. Platelet aggregation was expressed as the percentage of transmittance, considering 100% response the aggregation induced by ADP or collagen alone.

### 4.14. Statistical Analysis

Significant differences between the mean values of experimental groups were assessed by one-way ANOVA, and a Tukey post-test was used in order to compare all pairs of means. Values of *p* lower than 0.05 were considered significant.

## Figures and Tables

**Table 1:** Amino acid sequence of peptides obtained by digestion with trypsin, chymotrypsin or Glu-C endoproteinases from Peak 1 and Peak 2 of RP-HPLC, as determined by mass spectrometry (MALDI-TOF-TOF or nESI-MS/MS) or Edman *N*-terminal sequencing.

Peptide Sequence	HPLC Peak	*m*/*z*	z	Confidence ^a^	Error ^b^ (ppm)	Method ^c^	Domain
YIELVIVADHR	1	1327.8	1	MV	47.4	MALDI/DE NOVO	metalloproteinase
NLTPEEQRY	1	1149.6	1	MV	40.0	MALDI/DE NOVO	metalloproteinase
SRYHFVANR	1	1149.6	1	MV	7.8	MALDI/DE NOVO	metalloproteinase
YHFVANR	1	-		MV	-	EDMAN	metalloproteinase
MAHELGHNLGIHQDR	1	-	-	MV	-	EDMAN	metalloproteinase
MAHELGHNLGIHQDR	1	1727.8	1	MV	22.5	MALDI/DE NOVO	metalloproteinase
MAHELGHNLGLHQDR	1	576.6180	3	99	0.2	nESI/DE NOVO	metalloproteinase
DSCSCGSNSCIMSATVSNEPSSR	1	2493.0	1	MV	10.0	MALDI/DE NOVO	metalloproteinase
CIDNEPLR	1	1016.5	1	MV	16.7	MALDI/DE NOVO	metalloproteinase
NEPLRTDIVSPPFCGNYYPE *	1	2368.3	1	MV	88.2	MALDI/DE NOVO	metalloproteinase/disintegrin
LTTGSQCAEGLCCDQCR	1	2015.9	1	MV	47.6	MALDI/DE NOVO	disintegrin
FEGLCCDQCR	1	1344.4	1	MV	84.0	MALDI/DE NOVO	disintegrin
RTDIVSPPF **	1	1031.6	1	MV	46.5	MALDI/DE NOVO	disintegrin
YIELVIVADHR	2	1327.8	1	MV	47.4	MALDI/DE NOVO	metalloproteinase
VTLSADDTLDLFGTWR	2	-		MV	-	EDMAN	metalloproteinase
VALIGLEIWSSGELSK	2	1702.0	1	MV	34.0	MALDI/DE NOVO	metalloproteinase
YHFVANR	2	906.5	1	MV	46.3	MALDI/DE NOVO	metalloproteinase
MAHDLGHNLCIDHDR	2	1803.9	1	MV	54.8	MALDI/DE NOVO	metalloproteinase
MAHDLGHNLCLDHDR	2	902.4039	2	99	−0.4	nESI/DE NOVO	metalloproteinase
MAHDLGHNLCLDHDDR	2	959.9163	2	99	−1.5	nESI/DE NOVO	metalloproteinase
MAHELGHNLGLHQDDR	2	614.9611	3	99	1.2	nESI/DE NOVO	metalloproteinase
MAHELGHNLGLHQDR	2	864.4214	2	99	−2.1	nESI/DE NOVO	metalloproteinase
NKPSETDIVSPPVCGNYY **	2	2040.1	1	MV	79.4	MALDI/DE NOVO	metalloproteinase/disintegrin
CNGISAGCPRNPF **	2	1449.7	1	MV	44.1	MALDI/DE NOVO	disintegrin
RARGDDVNDYCNGISAGCPRNPFH *	2	2748.4	1	MV	68.7	MALDI/DE NOVO	disintegrin
ARGDDVNDYCNGISAGCPR	2	2097.1	1	MV	101.5	MALDI/DE NOVO	disintegrin
ARGDDVDDYCDGLDAGCPR	2	709.6204	3	91	−1.7	nESI/DE NOVO	disintegrin
AATCKLTTGSQCADGLCCDQCKFMRE *	2	3068.5	1	MV	71.0	MALDI/DE NOVO	disintegrin

^a^ Confidence: MALDI/DE NOVO and EDMAN (MV, manual validation); nESI/DE NOVO (PEAKS 8 De Novo average local confidence); ^b^ Error: error of the precursor ion (in ppm) relative to the proposed sequence; ^c^ Method: MALDI/DE NOVO: MALDI-TOF-TOF and manual de novo sequencing; EDMAN: *N*-terminal sequencing; nESI/DE NOVO: nLC-nanoelectrospray MS/MS and automatic de novo sequencing using PEAKS 8 software; * peptides generated by Glu-C endoproteinase digestion; ** peptides generated by chymotrypsin digestion. Peptides without asterisks were obtained following trypsin digestion. All cysteines are carboxamidomethylated. Sequences colored in red correspond to the mutated catalytic site.

## Supplementary Materials

The following are available online at www.mdpi.com/2072-6651/8/10/292/s1, Figure S1: High-resolution fragmentation spectrum of the *m*/*z* 576.6180 ion (*z* = 3; precursor error 0.2 ppm) from HPLC Peak 1 obtained on a QExactive Plus instrument (Thermo Scientific, Waltham, MA, USA) (**A**); the peptide sequence was interpreted by the PEAKS 8 de novo sequencing software. The average local confidence was set to 99%, and the local confidence score for each amino acid (i.e., “the likelihood of each amino acid assignment”) was ≥98%. (**B**) Ion table showing the calculated mass of possible fragment ions. All fragment ions matching a peak within the mass error tolerance were colored blue (b-ion series) or red (y ion series). The lower graph shows the mass error distribution of all matched fragment ions (represented by blue and red dots); Figure S2: High-resolution fragmentation spectrum of the *m*/*z* 614.9611 ion (*z* = 3; precursor error 1.2 ppm) from HPLC Peak 2 obtained on a QExactive Plus instrument (**A**). The peptide sequence was interpreted by the PEAKS 8 de novo sequencing software. The average local confidence was set to 99%, and the local confidence score for each amino acid (i.e., “the likelihood of each amino acid assignment”) was ≥98%. (**B**) Ion table showing the calculated mass of possible fragment ions. All fragment ions matching a peak within the mass error tolerance were colored blue (b-ion series) or red (y ion series). The lower graph shows mass error distribution of all matched fragment ions (represented by blue and red dots); Figure S3: High-resolution fragmentation spectrum of the *m*/*z* 864.4214 ion (*z* = 2; precursor error −2.1 ppm) from HPLC Peak 2 obtained on a QExactive Plus instrument (**A**). The peptide sequence was interpreted by the PEAKS 8 de novo sequencing software. The average local confidence was set to 99%, and the local confidence score for each amino acid (i.e., “the likelihood of each amino acid assignment”) was ≥98%. (**B**) Ion table showing the calculated mass of possible fragment ions. All fragment ions matching a peak within the mass error tolerance were colored blue (b-ion series) or red (y ion series). The lower graph shows mass error distribution of all matched fragment ions (represented by blue and red dots); Figure S4: High-resolution fragmentation spectrum of the *m*/*z* 959.9163 ion (*z* = 2; precursor error −1.5 ppm) from HPLC Peak 2 obtained on a QExactive Plus instrument (**A**). The peptide sequence was interpreted by the PEAKS 8 de novo sequencing software. The average local confidence was set to 99%, and the local confidence score for each amino acid (i.e., “the likelihood of each amino acid assignment”) was ≥ 98%. (**B**) Ion table showing the calculated mass of possible fragment ions. All fragment ions matching a peak within the mass error tolerance were colored blue (b-ion series) or red (y ion series). The lower graph shows mass error distribution of all matched fragment ions (represented by blue and red dots); Figure S5: High-resolution fragmentation spectrum of the *m*/*z* 902.4039 ion (*z* = 2; precursor error −0.4 ppm) from HPLC Peak 2 obtained on a QExactive Plus instrument (**A**). The peptide sequence was interpreted by the PEAKS 8 de novo sequencing software. The average local confidence was set to 99%, and the local confidence score for each amino acid (i.e., “the likelihood of each amino acid assignment”) was ≥98%. (**B**) Ion table showing the calculated mass of possible fragment ions. All fragment ions matching a peak within the mass error tolerance were colored blue (b-ion series) or red (y ion series). The lower graph shows mass error distribution of all matched fragment ions (represented by blue and red dots); Figure S6: High-resolution fragmentation spectrum of the *m*/*z* 709.6204 ion (*z* = 3; precursor error −1.7 ppm) from HPLC Peak 2 obtained on a QExactive Plus instrument (**A**). The peptide sequence was interpreted by the PEAKS 8 de novo sequencing software. The average local confidence was 91%, and the local confidence score for each amino acid (i.e., “the likelihood of each amino acid assignment”) is shown in the yellow inset. (**B**) Ion table showing the calculated mass of possible fragment ions. All fragment ions matching a peak within the mass error tolerance were colored blue (b-ion series) or red (y ion series). The lower graph shows mass error distribution of all matched fragment ions (represented by blue and red dots); Figure S7: Sequence coverage of mature BlatPII-c showing de novo sequenced tryptic peptides from HPLC Peak 1 with average local confidence (ALC) ≥99%, according to PEAKS 8 De Novo software. MS data were acquired using a Q Exactive Plus high resolution instrument. The similarity-based alignment was performed by the PepExplorer software, using the following search parameters: minimum identity = 75%; minimum number of residues per peptide = 6; substitution matrix = PAM30MS. Under these conditions, the sequence coverage obtained for BlatPII-c was 22.1%; Figure S8: Sequence coverage of mature BlatPII-c showing de novo sequenced tryptic peptides from HPLC Peak 2 with average local confidence (ALC) ≥99%, according to PEAKS 8 De Novo software. MS data were acquired using a Q Exactive Plus high resolution instrument. The similarity-based alignment was performed by the PepExplorer software, using the following search parameters: minimum identity = 75%; minimum number of residues per peptide = 6; substitution matrix = PAM30MS. Under these conditions, the sequence coverage obtained for BlatPII-c was 49.1%.
