# Supplementary material for: Novel Catalytically-Inactive PII Metalloproteinases from a Viperid Snake Venom with Substitutions in the Canonical Zinc-Binding Motif
Source: Toxins (Basel). 2016 Oct 12;8(10):292. doi: 10.3390/toxins8100292 (PMC5086652; doi:10.3390/toxins8100292)
Supplement: Supplementary file 1 [file toxins-08-00292-s001.pdf]

Supplementary Materials: Novel Catalytically-Inactive PII Metalloproteinases from a Viperid Snake Venom with Substitutions in the Canonical Zinc-Binding Motif

Erika Camacho , Libia Sanz, Teresa Escalante, Alicia Pérez, Fabián Villalta, Bruno Lomonte, Ana Gisele C. Neves-Ferreira , Andrés Feoli, Juan J. Calvete, José María Gutiérrez and Alexandra Rucavado

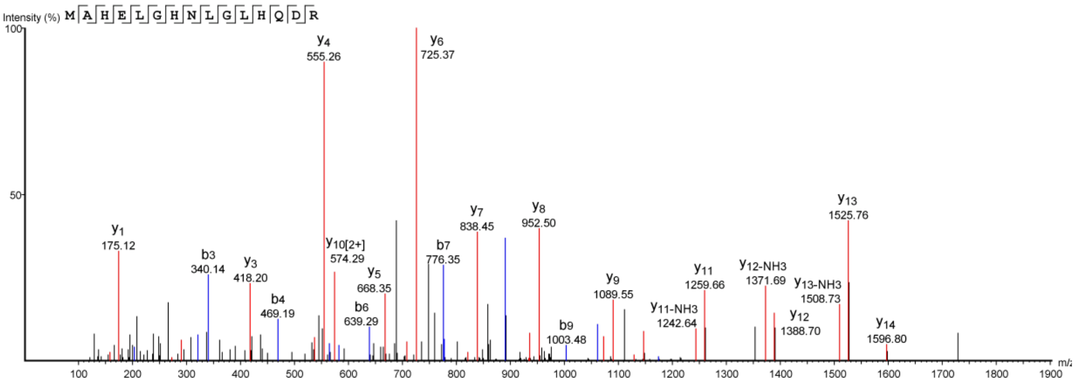

(A)

#	Immonium	b	b-H2O	b-NH3	b (2+)	Seq	y	y-H2O	y-NH3	y (2+)	#
1	104.05	132.05	114.04	115.02	66.52	M					15
2	44.05	203.08	185.07	186.06	102.04	A	1596.80	1578.79	1579.77	798.90	14
3	110.07	340.14	322.13	323.12	170.57	H	1525.76	1507.75	1508.73	763.38	13
4	102.06	469.19	451.18	452.16	235.09	E	1388.70	1370.69	1371.69	694.85	12
5	86.10	582.27	564.26	565.26	291.64	L	1259.66	1241.65	1242.64	630.33	11
6	30.03	639.29	621.28	622.27	320.15	G	1146.58	1128.56	1129.55	573.79	10
7	110.07	776.35	758.34	759.32	388.68	H	1089.55	1071.54	1072.53	545.28	9
8	87.06	890.39	872.38	873.37	445.70	N	952.50	934.49	935.47	476.75	8
9	86.10	1003.48	985.47	986.45	502.24	L	838.45	820.44	821.43	419.73	7
10	30.03	1060.50	1042.49	1043.47	530.75	G	725.37	707.36	708.34	363.18	6
11	86.10	1173.59	1155.57	1156.56	587.29	L	668.35	650.34	651.32	334.67	5
12	110.07	1310.64	1292.63	1293.62	655.82	H	555.26	537.25	538.24	278.13	4
13	101.07	1438.70	1420.69	1421.67	719.85	Q	418.20	400.19	401.18	209.60	3
14	88.04	1553.73	1535.72	1536.70	777.35	D	290.14	272.14	273.12	145.57	2
15	129.11					R	175.12	157.11	158.09	88.06	1

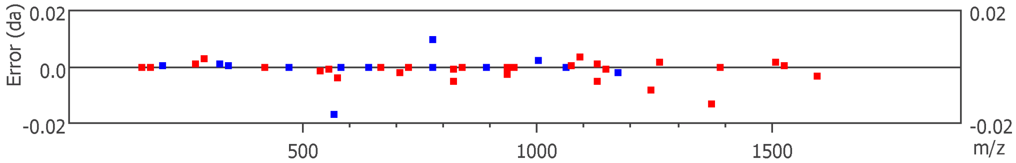

(B)

Figure S1. High-resolution fragmentation spectrum of the *m*/*z* 576.6180 ion (*z* = 3; precursor error 0.2 ppm) from HPLC Peak 1 obtained on a QExactive Plus instrument (Thermo Scientific, Waltham, MA, USA) (**A**); the peptide sequence was interpreted by the PEAKS 8 de novo sequencing software. The average local confidence was set to 99%, and the local confidence score for each amino acid (i.e., "the likelihood of each amino acid assignment") was ≥98%. (**B**) Ion table showing the calculated mass of possible fragment ions. All fragment ions matching a peak within the mass error tolerance were colored blue (b-ion series) or red (y ion series). The lower graph shows the mass error distribution of all matched fragment ions (represented by blue and red dots).

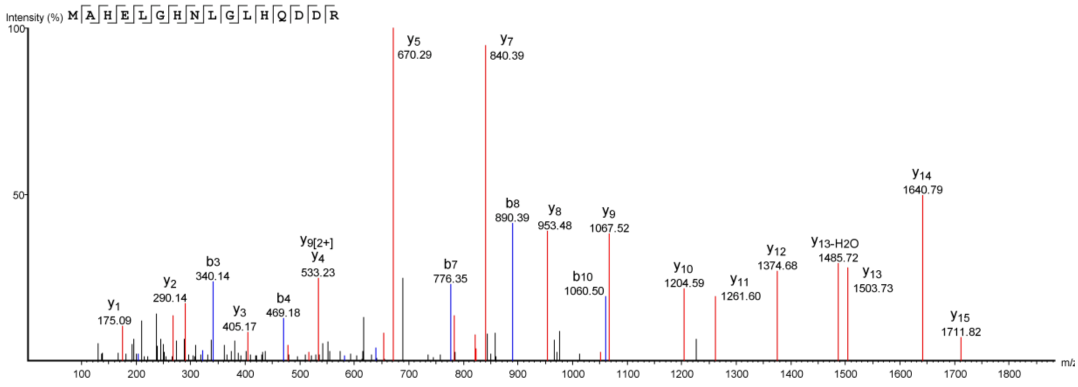

(A)

#	Immonium	b	b-H2O	b-NH3	b (2+)	Seq	y	y-H2O	y-NH3	y (2+)	#
1	104.05	132.05	114.04	115.02	66.52	M					16
2	44.05	203.08	185.07	186.06	102.04	A	1711.82	1693.82	1694.80	856.41	15
3	110.07	340.14	322.13	323.12	170.57	H	1640.79	1622.78	1623.76	820.90	14
4	102.06	469.18	451.18	452.16	235.09	E	1503.73	1485.72	1486.70	752.36	13
5	86.10	582.28	564.26	565.24	291.64	L	1374.68	1356.68	1357.66	687.84	12
6	30.03	639.29	621.28	622.27	320.15	G	1261.60	1243.59	1244.58	631.30	11
7	110.07	776.35	758.34	759.32	388.68	H	1204.59	1186.57	1187.55	602.79	10
8	87.06	890.39	872.38	873.37	445.70	N	1067.52	1049.51	1050.50	534.24	9
9	86.10	1003.48	985.47	986.45	502.24	L	953.48	935.47	936.45	477.24	8
10	30.03	1060.50	1042.49	1043.47	530.75	G	840.39	822.40	823.37	420.70	7
11	86.10	1173.58	1155.57	1156.56	587.29	L	783.37	765.36	766.35	392.19	6
12	110.07	1310.64	1292.63	1293.62	655.82	H	670.29	652.28	653.26	335.65	5
13	101.07	1438.70	1420.69	1421.67	719.85	Q	533.23	515.22	516.22	267.11	4
14	88.04	1553.73	1535.72	1536.70	777.36	D	405.17	387.16	388.15	203.08	3
15	88.04	1668.76	1650.74	1651.73	834.88	D	290.14	272.14	273.12	145.57	2
16	129.11					R	175.12	157.11	158.09	88.06	1

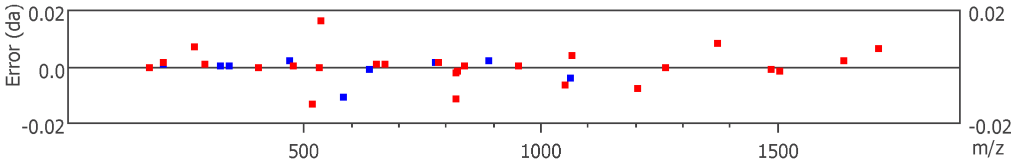

(B)

Figure S2. High-resolution fragmentation spectrum of the *m*/*z* 614.9611 ion (*z* = 3; precursor error 1.2 ppm) from HPLC Peak 2 obtained on a QExactive Plus instrument (**A**). The peptide sequence was interpreted by the PEAKS 8 de novo sequencing software. The average local confidence was set to 99%, and the local confidence score for each amino acid (i.e., "the likelihood of each amino acid assignment") was ≥98%. (**B**) Ion table showing the calculated mass of possible fragment ions. All fragment ions matching a peak within the mass error tolerance were colored blue (b-ion series) or red (y ion series). The lower graph shows the mass error distribution of all matched fragment ions (represented by blue and red dots).

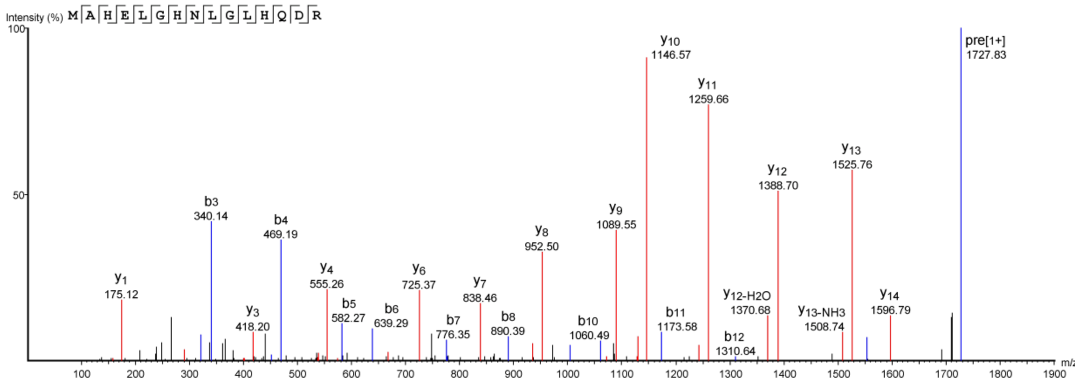

(A)

#	Immonium	b	b-H2O	b-NH3	b (2+)	Seq	y	y-H2O	y-NH3	y (2+)	#
1	104.05	132.05	114.04	115.02	66.52	M					15
2	44.05	203.09	185.07	186.06	102.04	A	1596.79	1578.79	1579.77	798.90	14
3	110.07	340.14	322.13	323.12	170.57	H	1525.76	1507.75	1508.74	763.38	13
4	102.06	469.19	451.17	452.16	235.09	E	1388.70	1370.68	1371.68	694.85	12
5	86.10	582.27	564.26	565.24	291.64	L	1259.66	1241.65	1242.63	630.33	11
6	30.03	639.29	621.28	622.27	320.15	G	1146.57	1128.55	1129.55	573.80	10
7	110.07	776.35	758.34	759.32	388.68	H	1089.55	1071.54	1072.53	545.28	9
8	87.06	890.39	872.38	873.37	445.70	N	952.50	934.49	935.47	476.75	8
9	86.10	1003.47	985.47	986.45	502.24	L	838.46	820.44	821.43	419.73	7
10	30.03	1060.49	1042.49	1043.47	530.75	G	725.37	707.36	708.34	363.18	6
11	86.10	1173.58	1155.57	1156.56	587.29	L	668.34	650.34	651.32	334.67	5
12	110.07	1310.64	1292.63	1293.62	655.82	H	555.26	537.26	538.24	278.13	4
13	101.07	1438.70	1420.69	1421.67	719.85	Q	418.20	400.19	401.18	209.60	3
14	88.04	1553.73	1535.72	1536.70	777.35	D	290.15	272.14	273.12	145.57	2
15	129.11					R	175.12	157.11	158.09	88.06	1

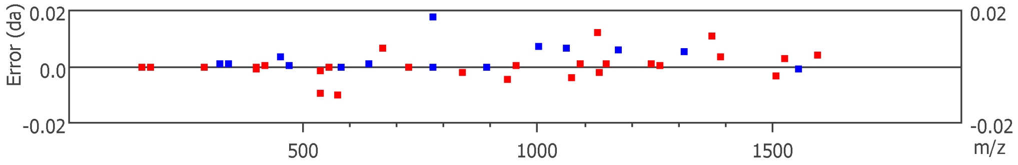

(B)

Figure S3. High-resolution fragmentation spectrum of the *m*/*z* 864.4214 ion (*z* = 2; precursor error −2.1 ppm) from HPLC Peak 2 obtained on a QExactive Plus instrument (**A**). The peptide sequence was interpreted by the PEAKS 8 de novo sequencing software. The average local confidence was set to 99%, and the local confidence score for each amino acid (i.e., "the likelihood of each amino acid assignment") was ≥98%. (**B**) Ion table showing the calculated mass of possible fragment ions. All fragment ions matching a peak within the mass error tolerance were colored blue (b-ion series) or red (y ion series). The lower graph shows the mass error distribution of all matched fragment ions (represented by blue and red dots).

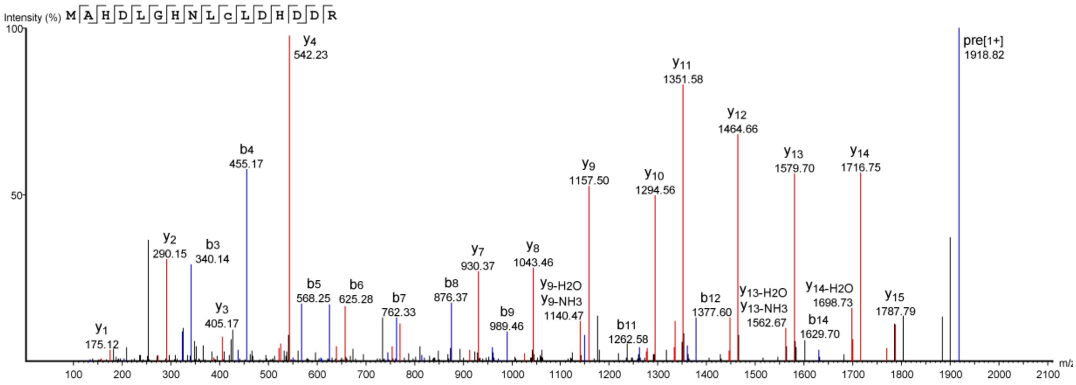

(A)

#	Immonium	b	b-H2O	b-NH3	b (2+)	Seq	y	y-H2O	y-NH3	y (2+)	#
1	104.05	132.05	114.04	115.02	66.52	M					16
2	44.05	203.08	185.07	186.06	102.04	A	1787.79	1769.77	1770.76	894.39	15
3	110.07	340.14	322.13	323.12	170.57	H	1716.75	1698.73	1699.73	858.88	14
4	88.04	455.17	437.16	438.14	228.09	D	1579.70	1561.67	1562.67	790.35	13
5	86.10	568.25	550.25	551.23	284.63	L	1464.66	1446.66	1447.64	732.83	12
6	30.03	625.28	607.27	608.26	313.14	G	1351.58	1333.57	1334.56	676.29	11
7	110.07	762.33	744.33	745.31	381.67	H	1294.56	1276.55	1277.54	647.78	10
8	87.06	876.37	858.37	859.35	438.69	N	1157.50	1139.48	1140.47	579.25	9
9	86.10	989.46	971.45	972.44	495.23	L	1043.46	1025.44	1026.43	522.23	8
10	133.04	1149.48	1131.48	1132.47	575.25	C(+57.02)	930.37	912.37	913.35	465.69	7
11	86.10	1262.58	1244.57	1245.55	631.79	L	770.34	752.33	753.33	385.67	6
12	88.04	1377.60	1359.59	1360.58	689.30	D	657.26	639.25	640.23	329.13	5
13	110.07	1514.66	1496.65	1497.64	757.83	H	542.23	524.22	525.21	271.62	4
14	88.04	1629.70	1611.68	1612.66	815.35	D	405.17	387.16	388.15	203.08	3
15	88.04	1744.72	1726.71	1727.69	872.86	D	290.15	272.14	273.12	145.57	2
16	129.11					R	175.12	157.11	158.09	88.06	1

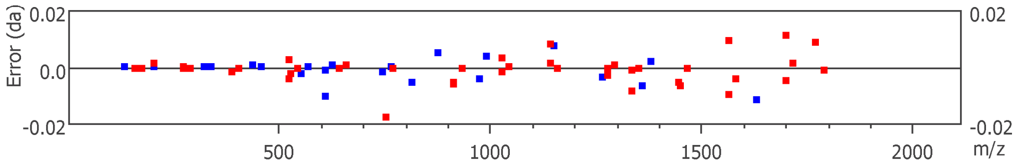

(B)

Figure S4. High-resolution fragmentation spectrum of the *m*/*z* 959.9163 ion (*z* = 2; precursor error −1.5 ppm) from HPLC Peak 2 obtained on a QExactive Plus instrument (**A**). The peptide sequence was interpreted by the PEAKS 8 de novo sequencing software. The average local confidence was set to 99%, and the local confidence score for each amino acid (i.e., "the likelihood of each amino acid assignment") was ≥ 98%. (**B**) Ion table showing the calculated mass of possible fragment ions. All fragment ions matching a peak within the mass error tolerance were colored blue (b-ion series) or red (y ion series). The lower graph shows the mass error distribution of all matched fragment ions (represented by blue and red dots).

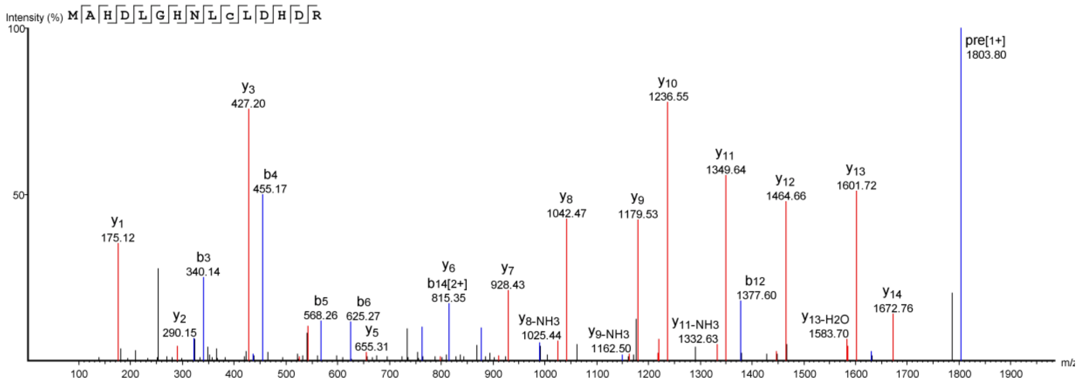

(**A**)

#	Immonium	b	b-H2O	b-NH3	b (2+)	Seq	y	y-H2O	y-NH3	y (2+)	#
1	104.05	132.05	114.04	115.02	66.52	M					15
2	44.05	203.09	185.07	186.06	102.04	A	1672.76	1654.75	1655.73	836.88	14
3	110.07	340.14	322.13	323.12	170.57	H	1601.72	1583.70	1584.70	801.36	13
4	88.04	455.17	437.16	438.14	228.09	D	1464.66	1446.65	1447.65	732.83	12
5	86.10	568.26	550.24	551.23	284.63	L	1349.64	1331.63	1332.63	675.32	11
6	30.03	625.27	607.27	608.25	313.14	G	1236.55	1218.54	1219.53	618.78	10
7	110.07	762.34	744.33	745.31	381.67	H	1179.53	1161.52	1162.50	590.27	9
8	87.06	876.38	858.37	859.35	438.69	N	1042.47	1024.46	1025.44	521.74	8
9	86.10	989.46	971.45	972.44	495.23	L	928.43	910.42	911.40	464.72	7
10	133.04	1149.50	1131.48	1132.47	575.25	C(+57.02)	815.35	797.34	798.32	408.17	6
11	86.10	1262.58	1244.57	1245.55	631.79	L	655.31	637.31	638.29	328.16	5
12	88.04	1377.60	1359.59	1360.58	689.30	D	542.23	524.22	525.21	271.62	4
13	110.07	1514.66	1496.65	1497.64	757.83	H	427.20	409.19	410.18	214.10	3
14	88.04	1629.71	1611.68	1612.66	815.35	D	290.15	272.14	273.12	145.57	2
15	129.11					R	175.12	157.11	158.09	88.06	1

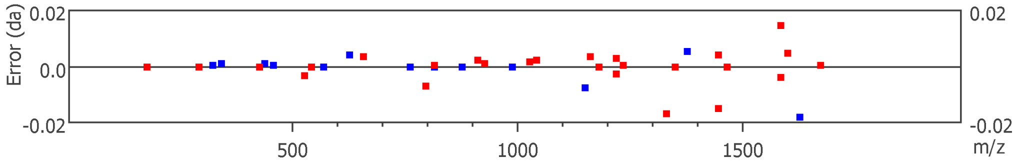

(**B**)

Figure S5. High-resolution fragmentation spectrum of the *m/z* 902.4039 ion ($z = 2$; precursor error −0.4 ppm) from HPLC Peak 2 obtained on a QExactive Plus instrument (**A**). The peptide sequence was interpreted by the PEAKS 8 de novo sequencing software. The average local confidence was set to 99%, and the local confidence score for each amino acid (i.e., "the likelihood of each amino acid assignment") was ≥98%. (**B**) Ion table showing the calculated mass of possible fragment ions. All fragment ions matching a peak within the mass error tolerance were colored blue (b-ion series) or red (y ion series). The lower graph shows the mass error distribution of all matched fragment ions (represented by blue and red dots).

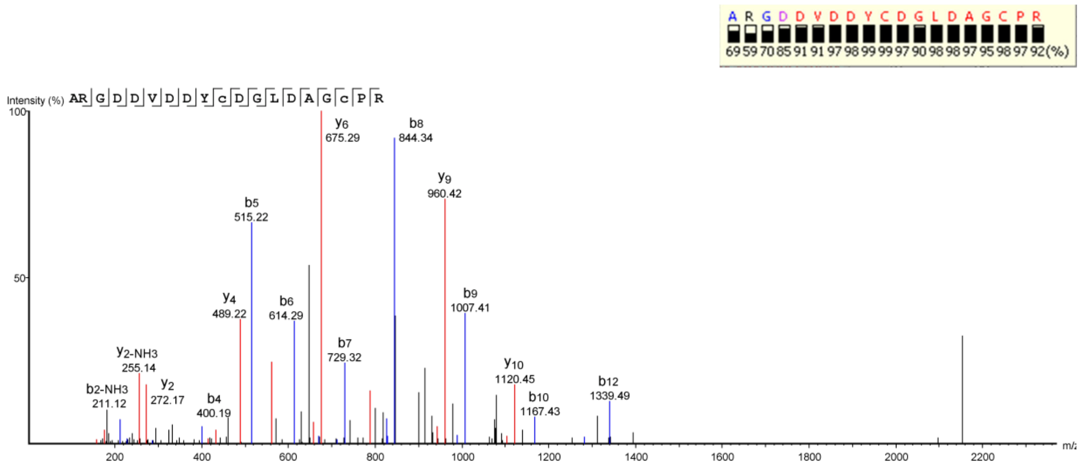

(**A**)

#	Immonium	b	b-H2O	b-NH3	b (2+)	Seq	y	y-H2O	y-NH3	y (2+)	#
1	44.05	72.04	54.03	55.02	36.52	A					19
2	129.11	228.14	210.14	211.12	114.57	R	2055.81	2037.80	2038.79	1028.41	18
3	30.03	285.16	267.16	268.14	143.08	G	1899.71	1881.70	1882.68	950.36	17
4	88.04	400.19	382.18	383.17	200.60	D	1842.69	1824.68	1825.66	921.85	16
5	88.04	515.22	497.21	498.19	258.11	D	1727.66	1709.65	1710.64	864.33	15
6	72.08	614.29	596.28	597.26	307.64	V	1612.64	1594.63	1595.61	806.82	14
7	88.04	729.32	711.31	712.29	365.16	D	1513.57	1495.56	1496.54	757.28	13
8	88.04	844.34	826.33	827.33	422.67	D	1398.54	1380.53	1381.51	699.77	12
9	136.08	1007.41	989.39	990.38	504.20	Y	1283.51	1265.50	1266.49	642.26	11
10	133.04	1167.43	1149.43	1150.41	584.22	C(+57.02)	1120.45	1102.44	1103.43	560.73	10
11	88.04	1282.47	1264.45	1265.44	641.73	D	960.42	942.41	943.40	480.71	9
12	30.03	1339.49	1321.48	1322.46	670.26	G	845.39	827.38	828.36	423.20	8
13	86.10	1452.57	1434.56	1435.54	726.79	L	788.37	770.36	771.34	394.69	7
14	88.04	1567.60	1549.59	1550.57	784.30	D	675.29	657.27	658.26	338.14	6
15	44.05	1638.63	1620.62	1621.61	819.82	A	560.26	542.25	543.23	280.63	5
16	30.03	1695.66	1677.65	1678.63	848.33	G	489.22	471.21	472.20	245.11	4
17	133.04	1855.69	1837.68	1838.66	928.34	C(+57.02)	432.20	414.19	415.18	216.60	3
18	70.07	1952.74	1934.73	1935.71	976.87	P	272.17	254.16	255.14	136.59	2
19	129.11					R	175.12	157.11	158.09	88.06	1

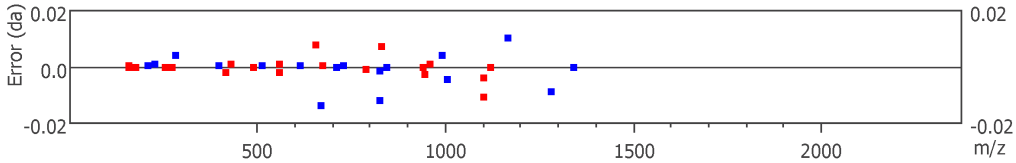

(**B**)

Figure S6. High-resolution fragmentation spectrum of the *m*/*z* 709.6204 ion (*z* = 3; precursor error −1.7 ppm) from HPLC Peak 2 obtained on a QExactive Plus instrument (**A**). The peptide sequence was interpreted by the PEAKS 8 de novo sequencing software. The average local confidence was 91%, and the local confidence score for each amino acid (i.e., "the likelihood of each amino acid assignment") is shown in the yellow inset. (**B**) Ion table showing the calculated mass of possible fragment ions. All fragment ions matching a peak within the mass error tolerance were colored blue (b-ion series) or red (y ion series). The lower graph shows the mass error distribution of all matched fragment ions (represented by blue and red dots).

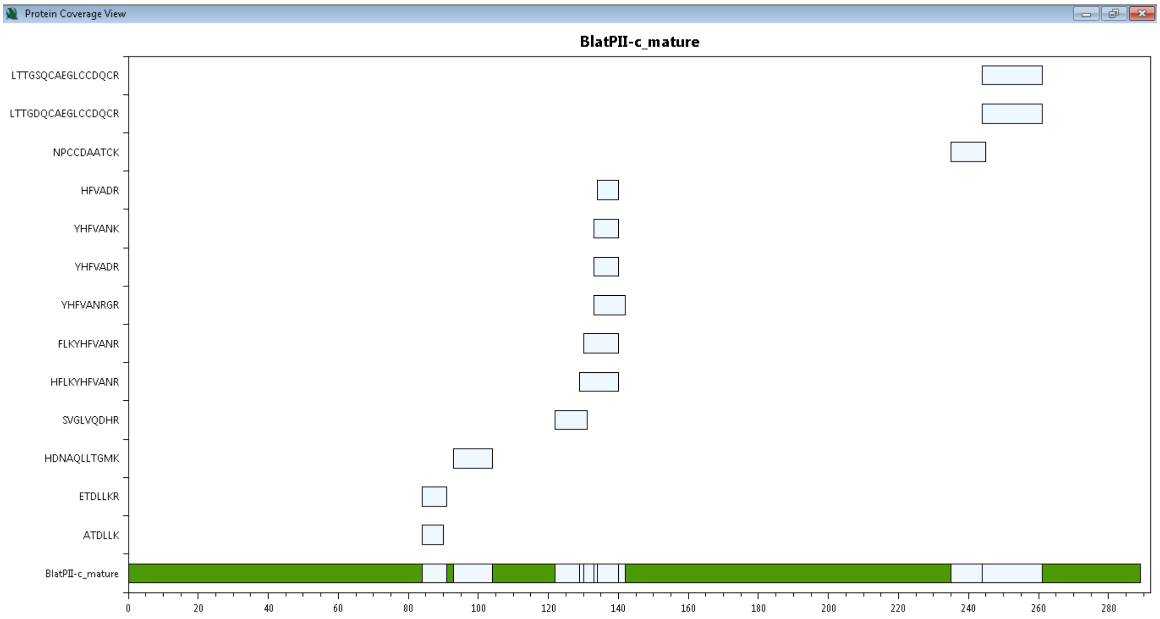

Figure S7. Sequence coverage of mature BlatPII-c showing de novo sequenced tryptic peptides from HPLC Peak 1 with average local confidence (ALC) ≥99%, according to PEAKS 8 De Novo software. MS data were acquired using a Q Exactive Plus high resolution instrument. The similarity-based alignment was performed by the PepExplorer software, using the following search parameters: minimum identity = 75%; minimum number of residues per peptide = 6; substitution matrix = PAM30MS. Under these conditions, the sequence coverage obtained for BlatPII-c was 22.1%.

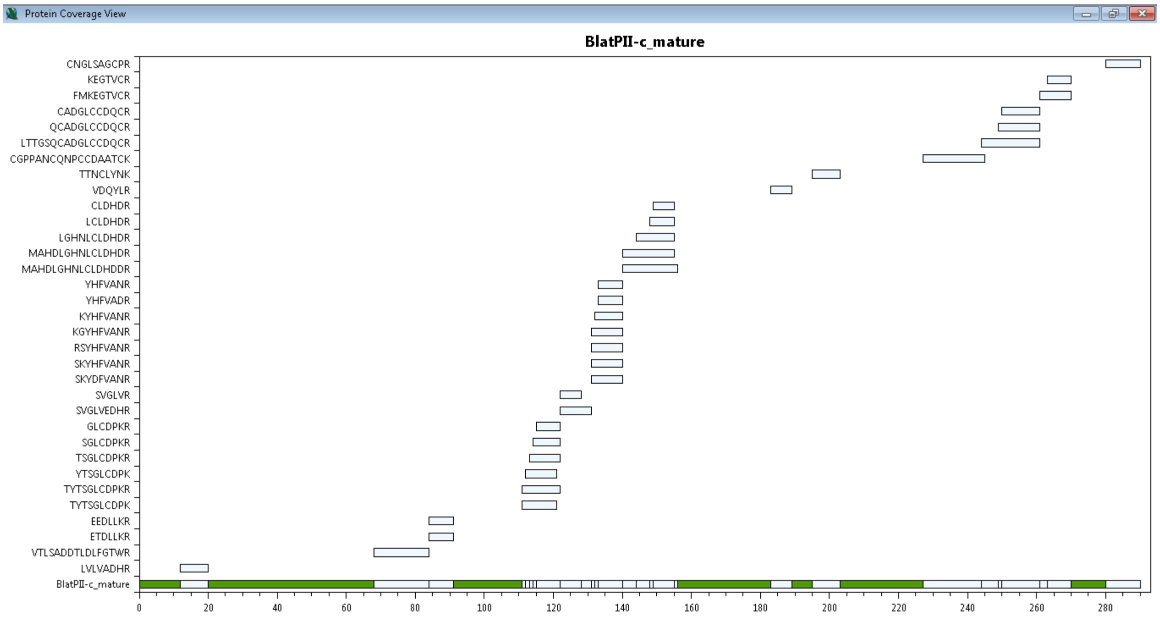

Figure S8. Sequence coverage of mature BlatPII-c showing de novo sequenced tryptic peptides from HPLC Peak 2 with average local confidence (ALC) ≥99%, according to PEAKS 8 De Novo software. MS data were acquired using a Q Exactive Plus high resolution instrument. The similarity-based alignment was performed by the PepExplorer software, using the following search parameters: minimum identity = 75%; minimum number of residues per peptide = 6; substitution matrix = PAM30MS. Under these conditions, the sequence coverage obtained for BlatPII-c was 49.1%.
